# *Valeriana officinalis* Dry Plant Extract for Direct Compression: Preparation and Characterization

**DOI:** 10.3797/scipharm.1206-05

**Published:** 2012-07-12

**Authors:** Loreana Gallo, María Veronica Ramírez-Rigo, Juliana Piña, Santiago Palma, Daniel Allemandi, Verónica Bucalá

**Affiliations:** 1 Department of Chemical Engineering - Universidad Nacional del Sur (UNS), PLAPIQUI (UNS-CONICET), Camino La Carrindanga Km. 7, (8000) Bahía Blanca, Argentina.; 2 Department of Pharmacy, Facultad de Ciencias Químicas, Universidad Nacional de Córdoba, CONICET, Edificio de Ciencias II, Ciudad Universitaria, (5000) Córdoba, Argentina.

**Keywords:** *Valeriana officinalis*, Dry plant extract, Hygroscopicity, Physico-mechanical properties, Direct compression, Glass transition temperature, Tablets

## Abstract

*Valeriana officinalis L.* (Valerianaceae) is one of the most widely used plants for the treatment of anxiety and insomnia. Usually dry plant extracts, including *V. officinalis*, are hygroscopic materials with poor physico-mechanical properties that can be directly compressed.

A *V. officinalis* dry extract with moderate hygroscocity is suitable for direct compression, and was obtained by using a simple and economical technique. The *V. officinalis* fluid extract was oven-dried with colloidal silicon dioxide as a drying adjuvant. The addition of colloidal silicon dioxide resulted in a dry plant extract with good physico-mechanical properties for direct compression and lower hygroscopicity than the dry extract without the carrier. The dry plant extract glass transition temperature was considerably above room temperature (about 72 °C). The colloidal silicon dioxide also produced an antiplasticizing effect, improving the powder’s physical stability.

The pharmaceutical performance of the prepared *V. officinalis* dry extract was studied through the design of tablets. The manufactured tablets showed good compactability, friability, hardness, and disintegration time. Those containing a disintegrant (Avicel PH 101) exhibited the best pharmaceutical performance, having the lowest disintegration time of around 40 seconds.

## Introduction

Pharmaceutical tablets are the principal dosage form for drug delivery, representing two-thirds of the global market [1]. The main reasons for their continued popularity are the ease of manufacture, convenience of dosing, and large storage stability in comparison with liquid and semi-solid formulations.

Direct compression of the active ingredient with adequate excipients is one of the most advantageous processes for tablet manufacture [2, 3]. However, drugs and excipients must demonstrate low hygroscopicity and good flowability and compactability for successful operation. Hygroscopicity plays an important role in particle-particle interactions and may contribute to poor powder flowability as well as negatively affect the material’s physical and chemical stability [4]. Appropriate flowability is essential to ensure homogeneous and rapid flow and, thus, uniformity of dose and weight during die-filling. Compactability is necessary for satisfactory tableting (i.e., the powder must remain in the compact form once the compression force is removed) [5].

In general, dry plant extracts are complex, amorphous, and viscous materials. Due to their high affinity for water vapour, dry plant extracts tend to be hygroscopic and tacky and have poor physico-mechanical properties [6]. Consequently, the addition of adequate excipients and/or the use of appropriate processing technologies prior to direct compression are necessary. Besides, phytomedicine tablets usually contain a high dose of the dry plant extract; thus, the amount of excipients that can be added becomes a critical issue in order to manufacture tablets of reasonable size [7, 8]. Therefore, pre-processing is practically mandatory to obtain dry plant extracts suitable for direct compression [9–11].

The development of novel materials and products based on plant extracts is relevant due to the extensive knowledge of the chemical, pharmacological, and clinical properties of herbal medicines [12] and the continuous expansion of the demand for natural products [13]. This is particularly the case for *Valeriana officinalis* L. extracts obtained from the roots and rhizomes of the plant [14, 15]. This herbal extract is widely used for the treatment of anxiety and insomnia, two common mental health problems in the general population and in primary care settings [16]. Sedative activity has been reported for the valerenic acids of the extract, being the substances often used as an indicator of quality [17, 18]. The *V. officinalis* dry plant extract is usually formulated in several pharmaceutical solid dosage forms, including immediate-release tablets, capsules, swellable pellets, and coated tablets. Coated tablets are frequently used to mask its unpleasant smell and taste [16, 19, 20]. However, the coating process may increase the tablet weight. Due to its swellable outer layer, swellable pellets facilitate swallowing without any unpleasant sensation in the mouth [21, 22]. Among all of the feasible dosage forms, tablets can be mass-produced at lower costs and processing times [23].

The pure dry pulverulent extract of *V. officinalis* has some stability problems because of its hygroscopic characteristics that make the manufacture of tablets difficult. To enhance the powder stability, Wills et al. [24] studied the storage of the material under recommended ambient conditions. Another reported approach, adopted by Merkel [25], was the granulation of a mixture containing one part of the commercial Valerian extract powder with two parts of skim milk by using a volatile organic solvent as a binder. The dry granules obtained were highly stable regarding hygroscopicity, but had poor flow properties. The authors did not deal with the improvement of these properties because the granules were not designed for the tablet’s preparation. Although these are valuable contributions, to the best of our knowledge there is a lack of work involving the development of a *V. officinalis* dry extract with good flowability, storage stability, and compactability.

Based on the above reasons, the main goal of this work was the production of a *V. officinalis* dry extract suitable for direct compression as well as the pharmaceutical-performance evaluation of the tablets specially designed.

## Experimental

### Materials

Roots and rhizomes of *V. officinalis* (Droguería Argentina, Argentine) and colloidal silicon dioxide (Cab-O-Sil®, Cabot Argentina, Argentine) were used in the form received from the supplier. Distilled water and ethanol 96% (Soria, Argentine) were employed for the preparation of the fluid plant extract.

Lactose monohydrate (DC Lactose: direct-compression grade available as granulated/agglomerated α-lactose monohydrate, containing small amounts of anhydrous lactose), dicalcium phosphate dihydrate (Emcompress), microcrystalline cellulose (Avicel PH101), and magnesium stearate were purchased from Droguería Saporiti (Argentine) and were used in the form received for the tablets formulations.

### Methods

Fluid plant extract preparation

*V. officinalis* roots and rhizomes were macerated for 72 hours at room temperature in an extractive solvent solution of alcohol and water (70:30) under constant stirring, and were then filtered. The plant material:extractive solvent ratio was 1kg:5l, according to the guidelines given by Andrews and Basu [26].

#### Solid residue (SR) assay

The SR content of the fluid plant extract was determined by evaporation of the solvent under reduced pressure, followed by drying of the residue in an oven at 50 °C to constant weight. The experiment was performed in triplicate. The SR was 6.2 ± 0.15 % (w/v).

#### V. officinalis dry plant extract production

Colloidal silicon dioxide was dispersed as the drying adjuvant in the fluid plant extract and the prepared dispersion was then oven-dried. The solid concentration of the obtained dispersion was 12.4 % (w/v), corresponding to the 1:1 colloidal silicon dioxide:SR ratio. Colloidal silicon dioxide is widely used in the pharmaceutical industry because, among other functions, it acts as an adsorbent, glidant, and disintegrant agent [27, 28]. The quantity of the carrier was selected based on previous studies performed with *Melissa officinalis*, *Cardus marianus*, *Peumus boldus,* and *Rhamnus purshiana*[29, 30]. The solvent was gradually evaporated at room temperature under constant stirring to maintain the homogenized dispersion. As soon as the mixture turned semisolid, the final solvent evaporation was performed in an oven at 50 °C until weight variation was not detected anymore. The temperature was selected according to the drying recommendations given in the American Herbal Pharmacopoeia [31]. The obtained powder was milled with mortar and pestle, and then screened through a mesh #70.

In addition, the fluid plant extract without the carrier was oven-drying for comparative purposes.

### Dry plant extract powder characterization

#### Angle of repose

The angle of repose was determined through the fixed funnel method. A predefined mass of powder was poured through a funnel, which was located at a fixed height above graph paper placed on a flat horizontal surface. The height (*h*) and radius (*r*) of the formed conical pile were measured. The inverse tangent of the *h/r* ratio is the angle of repose [32].

#### Hausner ratio

To determine the density of the dry plant extract, the powder was gently poured into a 10 cm^3^ graduated cylinder. The bulk density (*D_B_*) was calculated as the ratio between the weight (g) of the sample contained in the cylinder and the volume occupied (10 cm^3^). To establish the tap density (*D_T_*), the cylinder was tapped until no measurable change in volume was noticed. The compressibility of the powder was evaluated using the Hausner ratio [34], calculated as shown in Eq. 1:

Eq. 1. $$\text{Hausner}\;\text{ratio}=D_{T}/D_{B}$$

#### Compactability curve

The dry plant extract alone and with different common excipients, was compressed for 5 seconds in a hydraulic press (Delfabro) at different forces (10, 15, and 20 kN). The hardness of each compact was determined as the average of six measurements using a hardness tester (AVIC).

#### Moisture sorption

Samples of 100 mg of the dry plant extract and the dry extract without the carrier (in triplicate) were stored at 25 °C in closed recipients containing glycerine-water solutions, which were able to keep the relative humidity (RH) constant at different values depending on the glycerine-water ratio [35]. The samples were weighed periodically until a constant weight was noted, and then the moisture sorption for each RH was calculated.

#### Thermal analysis. Glass transition temperature determination

In order to study the glass transition temperature of dry plant extracts exposed at different RH, a differential scanning calorimeter (DSC, Pyris 1, Perkin Elmer) was used. Samples of 10 mg of the dry plant extract were placed in aluminum pans and scanned from 30 to 100 °C at a heating rate of 10 °C/min. The purpose of this first thermal scan was to eliminate the residual moisture that would interfere with the determination of the glass transition temperature. Then, the samples were cooled from 100 to 30 °C at a cooling rate of 10 °C/min and heated again from 30 to 100 °C at 10 °C/min. The glass transition temperature value was determined by the half *ΔCp* method [36].

#### Tablets preparation

The recommended daily dose of *V. officinalis* dry extracts ranges from 300 to 600 mg [18]. For the tablet design, two daily intakes were established. Different excipients, which are described below, were physically mixed with the dry plant extract for tablet formulation (Table 1). Avicel PH 101, Emcompress, and DC Lactose were particularly selected to study their influences on the dry plant extract’s physico-mechanical properties and on the tablets’ compactability, friability, and disintegration time.

Avicel PH 101 is one of the most widely used filler-binders for direct compression because of its excellent compactibility and disintegrant properties. This excipient has poor flow properties that can be improved by mixing it with other fillers of good flowability [3].

Emcompress is the main common inorganic salt used as a direct compression filler-binder due to its good flow properties. This excipient has relatively low compactibility and its tablets do not easily disintegrate because Emcompress is a water-insoluble powder. Therefore, it is important to add a disintegrant with an active mechanism such as swelling, for example Avicel PH 101 [3, 37].

Another commonly used excipient for direct compression is DC Lactose due to its good flowability. Compared to other filler-binders, DC Lactose exhibits relatively poor binding properties. In addition, it is frequently combined with Avicel PH 101 to improve its disintegration time [37, 38].

Magnesium stearate, usually used as a lubricant, was also included in the formulation to prevent sticking to tablet punches and dies during compression.

The corresponding dose of *V. officinalis* dry plant extract was blended with the selected excipients, except magnesium stearate, for 12 minutes by tumbling. Then, a given amount of magnesium stearate was added and further blended for 4 minutes. The powders were compacted in an excentric tablet press (SC1, Talleres Sanchez, Argentine) equipped with 13 mm circular edge punches.

#### Friability test

Tablet friability was measured as the percentage of weight loss of 11 tablets (6.5 g in weight) tumbled in a friabilator (Scout) [39]. After 5 minutes of rotation at 25 rpm, any loose dust was removed and the tablets were reweighted. The weight loss (%Friability) was calculated through the following equation:

Eq. 2. $$\%\text{Friability}=\frac{{\text{W}}_{\text{i}}-{\text{W}}_{\text{r}}}{{\text{W}}_{\text{i}}}\times 100$$

where *W_i_* and *W_r_* were the initial and final weights of all the tablets, respectively [35].

#### Disintegration time

The disintegration time, which is defined as the time necessary for the complete disintegration of tablets, was determined according to FNA [39] (Hanson disintegrator). The tests were carried out in 800 ml of distilled water at 37 ± 0.5 °C. For each formulation, six randomly selected tablets were tested.

## Results and Discussion

### Dry plant extract characterization

Table 2 summarizes the angle of repose, Hausner ratio, disintegration time, and %Friability for the dry plant extract and the three prepared formulations. The results corresponding to the tablet formulations will be discussed in the following sub-section.

The angle of repose and the Hausner ratio have long been used to characterize powder flowability. The flow property has been classified by the USP 30-NF 25 [33] in terms of the angle of repose and Hausner ratio as follows: excellent (25–30° and 1.00–1.11, respectively), good (31–35° and 1.12–1.18), fair (36–40° and 1.19–1.25), and poor (>41° and >1.26). Consequently, the *V. Officinalis* dry plant extract exhibited fair to good flow properties for direct compression. On the other hand, the dry extract without the carrier presented a rubbery physical appearance that made the determination of its physico-mechanical properties impossible.

**Fig.1.**
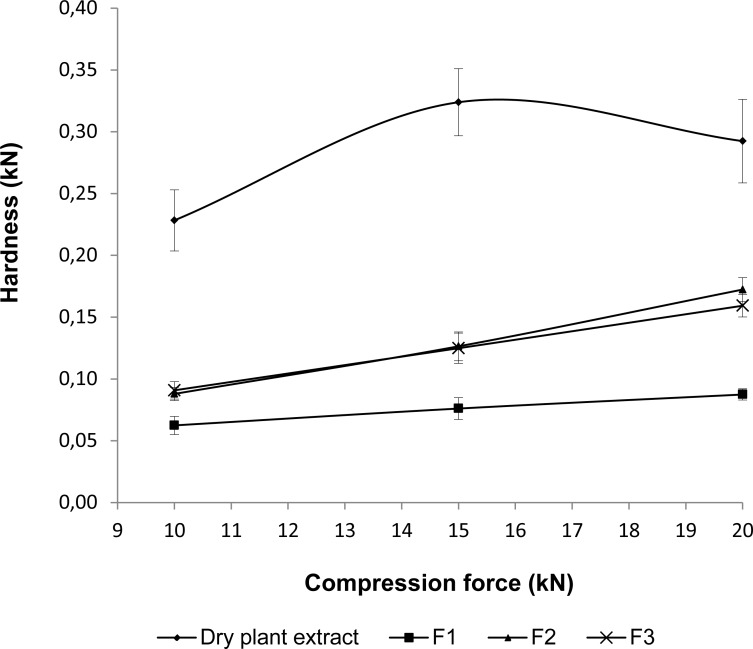
Compactibility curves for the dry plant extract and Formulations 1 to 3.

The *V. Officinalis* dry plant extract was a highly compactable material. In fact, very high hardness values (above 0.2 kN) were obtained after compaction at relatively low compression forces (See Figure 1). This behavior could be advantageous from a technical point of view. However, the biopharmaceutical performance of the *V. Officinalis* dry plant extract was unacceptable. Indeed, the disintegration time of tablets containing just the dry plant extract (see Table 2) was more than 20 minutes, which is the maximum allowable value for Valeriana tablets according to the USP 30-NF 25 [33].

**Fig. 2.**
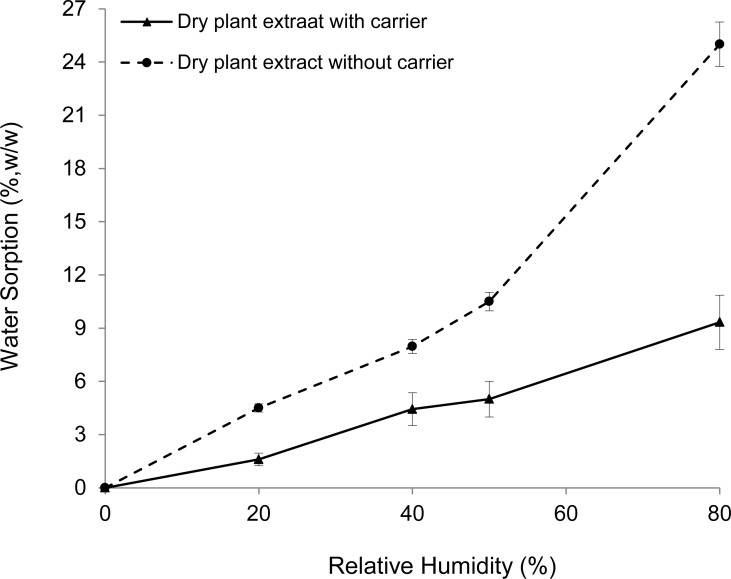
Water sorption of the dry plant extract with and without the carrier.

Generally, increasing the powder’s moisture content decreases its ability to flow adequately [41]. Figure 2 shows the water sorption of the dry plant extract and the dry extract without the drying adjuvant. According to the extent of water uptake after storage for one week at different RHs, Callahan et al. [42] proposed the following hygroscopicity classification: Class I, non-hygroscopic (no water sorption below 90% RH, and < 20% at 90% RH); Class II, slightly hygroscopic (no water sorption below 80% RH, and < 40% at 80% RH); Class III, moderately hygroscopic (< 5% below 60% RH, and < 50% at 80% RH); Class IV, very hygroscopic (> 5% below 60% RH). Therefore, the *V. officinalis* extract without the carrier was very hygroscopic (Class IV). In addition, and in agreement with reported data [20], the extract began to deliquesce when it was exposed to 40% RH. On the other hand, the dry plant extract behaved as a moderately hygroscopic material (Class III) and remained as a powder during the whole experiment, without exhibiting any apparent physical change. The plant extract was probably trapped within the solid particles of the colloidal silicon dioxide, being less available for water sorption [30].

**Fig. 3.**
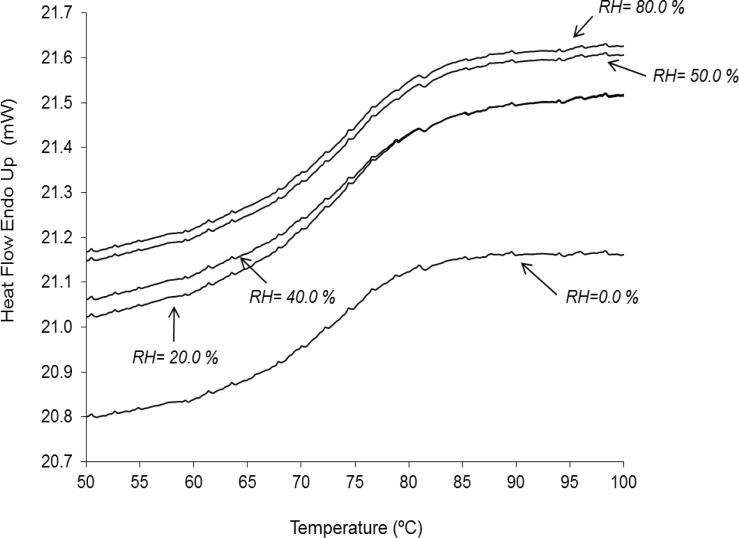
DSC thermograms showing glass transition temperatures of *V. officinalis* dry plant extract stored at different relative humidities.

Dried herbal extracts are amorphous materials that can exhibit a rubber-like state for temperatures higher than a certain critical value, known as the glass transition temperature [43]. For this reason, dried herbal extracts become sticky at room temperatures above the glass transition. Moreover, water can act as a plasticizer enabling the mobilization of amorphous components and, thus, decreases the material glass transition point to be below the storage temperature. Figure 3 presents the glass transition temperatures for *V. officinalis* dry plant extracts stored at different RHs. As it can be seen, the glass transition point was always well above room temperature and almost constant (about 72 °C). In contrast, the dry extract without the carrier showed rubbery characteristics at room temperature. Hence, it can be concluded that the addition of colloidal silicon dioxide provided an antiplasticizing effect, improving the *V. officinalis* extract storage stability.

### Design and pharmaceutical evaluation of tablets formulations

The addition of Emcompress, DC Lactose, and/or Avicel PH 101 in different proportions led to tablets harder than those containing just the *V. officinalis* dry plant extract (Fig. 1). The tablets’ hardness was satisfactory for the three proposed formulations. In fact, it was above 0.03 kN (i.e., the minimum allowable value) even at the lowest compression force.

The disintegration time was directly related to the formula composition. The formulations containing Avicel PH 101 presented fast disintegration (about 40 and 45 seconds for Formulations 3 and 2, respectively). On the other hand, a higher disintegration time which was close to the minimum acceptable one was found in the absence of Avicel PH 101 (Formulation 1, Table 2). As it was previously mentioned, Avicel PH 101 facilitated the rupture of tablets by swelling during water sorption.

The friability test has been designed to evaluate the ability of tablets to withstand abrasion during the tablets’ post-processing, handling, packaging, and shipping. As indicated by the results (Table 2), all of the formulations exhibited weight loss values lower than 1%, thus meeting the USP 30-NF 25 Pharmacopoeia requirements.

According to the USP 30-NF 25 [33], the three designed formulations exhibited fair to good flow properties for direct compression (Table 2), with the angle of repose and the *Hausner ratio* values similar to those obtained for the dry plant extract alone. This was an expected behavior considering that the dry plant extract and the selected excipients had good flowability and compactability.

In summary, the developed *V. officinalis* dry plant extract makes a widely demanded and accepted active ingredient available, with good flow properties for the manufacture of phytomedicine tablets by direct compression. Moreover, an adequate pharmaceutical performance is demonstrated through the design of different tablet formulations by the addition of common excipients that were rationally selected.

## Conclusions

The use of colloidal silicon dioxide as the carrier provided a *V. officinalis* dry plant extract with good flow and compactability properties. In addition, the developed dry plant extract was moderately hygroscopic in comparison with the highly hygroscopic dry extract without the carrier. The presence of colloidal silicon dioxide in the dry plant extract imparted an antiplasticizing effect that resulted in an almost constant glass transition temperature (≈ 72 °C). Moreover, and due to the considerably high glass transition temperature with respect to the room temperature, the obtained *V. officinalis* dry plant extract can be considered as a stable powder.

The proposed preparation method represents a simple and economic technological alternative for improving the *V. officinalis* dry extract’s hygroscopicity and physico-mechanical properties.

The excessive compactability exhibited by the dry plant extract required the addition of different excipients for a proper final formulation. The proposed tablet compositions, which were designed on a rational selection of excipients with different functionalities, allowed the demonstration of the potential use of the *V. officinalis* dry plant extract as an active phytomedicine ingredient. Indeed, the incorporation of the selected excipients led to tablets that fulfilled the pharmacopoeia requirements. The inclusion of a disintegrant (Avicel PH 101) provided tablets with low disintegration times and, thus, with the best pharmaceutical performance.

## Figures and Tables

**Tab. 1.** Composition of tablet formulations containing *V. officinalis* dry plant extract.

	**Formulation 1**	**Formulation 2**	**Formulation 3**
*V. officinalis dry plant extract*	300 mg	300 mg	300 mg
Avicel PH 101	–	146 mg	146 mg
DC Lactose	146 mg	–	146 mg
Emcompress	146 mg	146 mg	–
Mg stearate	8 mg	8 mg	8 mg
Total weight	600 mg	600 mg	600 mg

**Tab. 2.** Physico-mechanical and pharmaceutical properties. *V. officinalis* dry plant extract and proposed tablet formulations.

	**Angle of repose (°)^a^**	**Hausner ratio^b^**	**Disintegration time**	**Friability(%)**
*V. officinalis* dry plant extract	31 ± 1	1.21 ± 0.05	180 min	-
Formulation 1	30 ± 1	1.20 ± 0.03	17 min.	0.55
Formulation 2	32 ± 2	1.21 ± 0.02	45 sec.	0.57
Formulation 3	33 ± 2	1.21 ± 0.03	40 sec.	0.60

a The values represent the mean of twenty determinations ± standard deviation;

b The values represent the mean of sixteen determinations ± standard deviation.
